# Machine learning models for identifying preterm infants at risk of cerebral hemorrhage

**DOI:** 10.1371/journal.pone.0227419

**Published:** 2020-01-15

**Authors:** Varvara Turova, Irina Sidorenko, Laura Eckardt, Esther Rieger-Fackeldey, Ursula Felderhoff-Müser, Ana Alves-Pinto, Renée Lampe

**Affiliations:** 1 Research Unit for Pediatric Neuroorthopedics and Cerebral Palsy of the Buhl-Strohmaier Foundation, Orthopedic Department, Klinikum Rechts der Isar, Technical University of Munich, München, Germany; 2 Chair of Mathematical Modelling, Mathematical Faculty, Technical University of Munich, Garching bei München, Germany; 3 Departments of Pediatrics and Neonatology, University Hospital Essen, University of Duisburg‐Essen, Essen, Germany; 4 Department of Pediatrics, Neonatology, Klinikum Rechts der Isar, Technical University of Munich, München, Germany; Western University, CANADA

## Abstract

Intracerebral hemorrhage in preterm infants is a major cause of brain damage and cerebral palsy. The pathogenesis of cerebral hemorrhage is multifactorial. Among the risk factors are impaired cerebral autoregulation, infections, and coagulation disorders. Machine learning methods allow the identification of combinations of clinical factors to best differentiate preterm infants with intra-cerebral bleeding and the development of models for patients at risk of cerebral hemorrhage. In the current study, a Random Forest approach is applied to develop such models for extremely and very preterm infants (23–30 weeks gestation) based on data collected from a cohort of 229 individuals. The constructed models exhibit good prediction accuracy and might be used in clinical practice to reduce the risk of cerebral bleeding in prematurity.

## Introduction

Intraventricular cerebral hemorrhage (IVH) is a frequent complication in preterm infants, affecting the nervous system and leading to impairments of musculoskeletal and cognitive functions, speech development and vision. In most cases, bleeding initiates in the so-called germinal matrix (GM)—the brain area densely penetrated by blood vessels. The GM is responsible for the formation of immature neuronal and glial cells and disappears by the 32nd week of gestation (WG). Many factors influence the onset of cerebral hemorrhage [1]. According to studies [2–4], the most important role is played by the deficient and immature cerebral autoregulation, leading to significant fluctuations in the cerebral blood flow (CBF), as a result of which the fragile, muscle-lacking blood vessels of the GM are destroyed. But also various inflammatory diseases that affect the deformability of red blood cells, as well as coagulation disorders were found to be associated with the occurrence of IVH [5, 6].

Although in recent decades the survival rate of preterm infants has increased significantly, the reported percentage of cerebral hemorrhage in neonates with gestational age of < 30 weeks and birth weight < 1500 g remains between 22% and 25% [7]. A promising direction towards preventing cerebral hemorrhage in newborns is the creation of machine learning (ML) models that are able, with a certain degree of probability, to identify patients at risk of developing a cerebral hemorrhage. By analyzing clinical records of important parameters in preterm infants, it is possible to retrospectively develop such prognostic models by finding the optimal combination of parameters that makes possible to separate preterm infants with, from those without, bleeding. Preliminary work on the association of reduced or elevated values of various clinical parameters, such as leukocyte (Leu) and thrombocyte (Thro) counts, hematocrit (Ht), C-reactive protein (CRP), arterial oxygen partial pressure (pO_2_), blood oxygen saturation (SaO_2_), arterial carbon dioxide partial pressure (pCO_2_), blood acidity (pH), and mean arterial pressure (MAP), with the occurrence of cerebral hemorrhage, as well as the presence of correlations between some of the parameters, was carried out by the authors in [8, 9].

ML-methods have been successfully used for diagnosis and prognosis of various diseases, including stroke (see, for example, [10]). The use of ML-methods in neonatology is scarce. Few studies elaborating ML-tools for prediction of survival of preterm neonates are currently available; see, for example, [11] describing ML-models for estimation of mortality risk of very preterm neonates. Recent works [12] elaborated ML-models for more precise assessment of gestational age and to improve identification of preterm neonates.

The present article addresses the development of ML-models to identify preterm infants at risk for a cerebral hemorrhage. Clinical data of a cohort of 229 extremely (with 23–26 WG) and very preterm infants (with 27–30 WG), collected retrospectively in two German clinics were used to train and test the models. The collected data served as an input for Random Forest method available in the R software package, to find good predictive features as well as the optimal combination of features from analyzed data and to develop ML-models that are able to predict cerebral hemorrhage in extremely and very preterm infants.

## Material and methods

Data were collected retrospectively from clinical records of 265 preterm infants who were treated in two German Neonatology departments. Data were anonymized. The project and its procedures were approved by the Ethics Committees in both clinics, the Ethics Committee of the University Hospital ‘rechts der Isar’, Technical University of Munich (Ref. 364/15), and the Ethics Committee of the Essen University Hospital, University Duisburg-Essen (Ref. 16–7284-BO).

Patients were divided into two groups: with cerebral hemorrhage (affected group) and without cerebral hemorrhage (control group). Deceased newborns without cerebral hemorrhage were excluded from the control group, whilst those with hemorrhage were matched with neonates of the affected group by age (gestation week) and weight. This criterion for matching was chosen to make both control and affected groups comparable in terms of the physiological and anatomical factors that critically determine cerebral blood flow. The latter is influenced by the number and size of cerebral blood vessels [9], being dependent on brain weight, as estimated from birth weight [13]. Furthermore, the size of the GM, as the origin of cerebral hemorrhages, depends also on the gestational age and birth weight [14]. The GM reaches its maximum size of 5% of total brain volume by the 22nd week of gestation and decreases steadily from the 23rd week of gestation onwards, disappearing generally by the 34th week gestation. After all exclusions and after matching, 118 patients with IVH (75 extremely preterm; 43 very preterm) and 111 controls (62 extremely preterm; 49 very preterm) remained in the analysis. Their obstetric data are given in **Table 1**.

**Table 1:** Obstetric characteristics of the cohort.

Parameter	No IVH (n = 111)	With IVH (n = 118)	p-value^a^
**Gestational age [weeks]**	26.6±2.1	26.3±2.0	0.19
**Birth weight [g]**	853±249	881±298	0.58
**Sex [%Male]**	43.2	56.4	0.06
**Multiple birth [%, e.g. twins and triplets]**	36.9	38.9	0.79
**Normal vaginal delivery [%]**	6.3	9.3	0.46
**Rupture of the amniotic sac [%]**	31.5	22.9	0.18

^a^p-value based on the two-sided Wilcoxon rank-sum test for the null hypothesis that the group means for the two groups are equal.

Note that the control group is 6% smaller than the affected one because it was not possible to find in the cohort a number of age- and weight-matched control infants equal to the affected ones. As a consequence, some controls were matched with two affected neonates of the same gestational age and weight. However, since there were generally more measurements for controls than for affected neonates, we decided to include all affected infants in the study. An exact matching between groups based on sex was also not possible because of the higher prevalence of IVH in males compared to females which could not be matched in the control group. This difference in the relative number of males between groups was nevertheless not statistically significant.

There is prior evidence of a difference in the incidence of IVH between extremely and very preterm infants: with 45% in extremely preterm infants [15] and 24% in very preterm infants [16]. Furthermore, in our previous study [8], significant differences in some clinical parameters were found between extremely and very preterm newborns. Therefore, in the current study the group of extremely preterm infants was analyzed separately from the group of very preterm infants.

IVH was diagnosed by cranial ultrasound examination. According to standard practice, cranial ultrasound is performed routinely on the 1st, 3rd, 7th, and 14th days and sometimes more frequently if there are clinical signs of, or if ultrasound examination indicates, pathology.

Data samples of neonates of the affected group used in the development of ML-model were those collected before hemorrhage. Selection of data samples was done according to the protocol shown in Table 2 as follows: clinical measurements of infants for whom IVH was diagnosed on the first day of life were not included in the analysis. In cases where IVH was diagnosed as having occurred on the second day of life, the recordings made on the first day of life were used. For infants for whom IVH was diagnosed on the 3rd day of life or afterwards, data samples were considered up to the day when the previous routine ultrasound examination took place (e.g., only 1st day of life when IVH was diagnosed on the 3rd day, and first 3 days of life when IVH was diagnosed on the 4th till the 7th day). For newborns of the control group, all available data samples were included in the analysis.

**Table 2:** Procedure adopted to select data samples of affected neonates to be input into the model. The procedure considers the day of occurrence of IVH.

Day of IVH diagnosis	1	2–3	4–7	8–14	15–21
**Number of days of infant’s life since birth for which data samples were included in the analysis**	0	1	3	7	14

It is worth mentioning that the number of measurement samples for controls is usually higher than for affected infants. The difference comparing to the affected group is due to the fact that only records taken before hemorrhage are used in the analysis of affected neonates. This difference is reflected in Table 3. As discussed later, our machine learning pipeline accounted for this imbalance by oversampling the affected group.

**Table 3:** Number of observation days and measurement samples per infant for the control and affected groups.

	No IVH	With IVH
**Max observation days**	13	14
**Mean number of days±SD**	9.8±0.8	2.7±2.8
**Max measurements per day**	8	9.2
**Mean number of measurements per day±SD**	2.4±1.4	2.4±1.5
**Total number of measurements**	2373	801

Clinical parameters were obtained from arterial and capillary blood samples and included pH, pCO_2_, pO_2_, Leu, Ht, Thro, CRP, as well as measurements of MAP, SaO_2_, and Apgar-Score values ​​at 1, 5 and 10 minutes after birth (A1, A5, A10). To have complete records at a given time point, rarer measurements of Leu, Ht, Thro, and CRP were time-matched (within one day) with the more frequent records of blood gas values (pH, pO_2_, pCO_2_) and of MAP and SaO_2_.

In addition to the clinical parameters mentioned, the values taken from clinical records, we included also cerebral blood flow (CBF) as an additional parameter to train and validate the model. CBF values used here correspond to simulated values, derived using a mathematical model developed by the authors in a previous study [9]. Briefly, this model describes mathematically the dependence of CBF on different anatomical and physiological variables, and was adapted from a pre-existing model for the adult brain [17] to the brain of preterm infants. As in [17], the cerebrovascular system is represented in the model in the form of 19 consecutively connected compartments: 9 arterial, 1 capillary, and 9 venous. Each compartment has its own number of vessels and blood vessel characteristics (length, diameter, pCO_2_-reactivity). At the capillary level, the presence of the GM with its characteristic features (higher vascular density, increased diameter and decreased length of vessels compared to other brain regions, and reduced vascular response to pCO_2_ fluctuations) and the number of vessels according to the child's age is accounted for by the parallel connection of its circulatory system with the rest of the brain capillaries. The resistance of this cerebral network is computed as the sum of resistances of 19 compartments and the total CBF is calculated according to Kirchhoff's law. Vessels’ reactivity (the ability to constrict or dilate depending on the pCO_2_ content in blood) is modeled by making the diameter of the vessels to depend on the deviation of the pCO_2_ level from some nominal value. The myogenic autoregulation mechanism (the change in the diameter of vessels with blood pressure fluctuations) is taken into account by adjusting the model in accordance with published CBF measurements in preterm infants (see [9] for details). Despite the complexity of the model, the mathematical simulation of CBF has been also implemented in a user-friendly software interface not requiring deep mathematical knowledge.

Clinical datasets of affected and control infants used to build and evaluate ML-models were input without distinguishing between individual persons. Only the presence/absence of IVH and the membership to the group of extremely or very preterm neonates was taken into account. The age at which clinical records were done was not considered in the model.

ML-models were developed using the R software package [18, 19]. The Random Forest (RF) method was applied. RF is considered particularly well suited when a large number of features is considered [20] (13 in our case). In addition, RF has the advantage of computing the importance of each feature in the classification process.

Data were randomly divided into a training set (90%) and an unseen validation set (10%).

The accuracy of the model in the training stage was estimated using repeated 10-fold cross validation with three repeats. This procedure involves randomly dividing the set of observations into 10 groups, or folds, of approximately equal size. Then the ML-model is trained on 9 folds and is tested on the last fold, such that each fold becomes test set once. The procedure is repeated three times and the results are averaged. The folds are selected randomly in each of the three repeats. To handle the above mentioned data imbalance, the option “SMOTE” (Synthetic Minority Oversampling Technique) [21] was adopted, that allows changing the frequency of different classes of the training data. Specifically, the function call

‘train(hemorr~., data = dataset, method = "rf", metric = "Accuracy", trControl = control)’

with the parameter ‘control’ being the output of the call

‘trainControl(method = "repeatedcv",number = 10,repeats = 3,sampling = "smote")’ was used.

The importance of features for each of the two groups was estimated using a standard R-function “varImp”. The standard Recursive Feature Elimination (RFE) method provided by the caret R package was used to select the number of features.

Two main metrics, the mean accuracy and the characteristic Cohen’s kappa (see Table 4), were used to characterize the quality of the model. In the validation stage, the model performance was estimated on unseen data by calculating the following metrics (Table 4): 1) the observed accuracy, 2) the no-information rate (a classifier that attributes a sample to a class with the probability equal to the class percentage in the data), 3) the sensitivity (the percentage correctly classified neonates with hemorrhage), and 4) the specificity (the percentage of correctly classified neonates without hemorrhage).

**Table 4:** Variables and metrics used in evaluation of model performance.

Metrics/Variable	Definition
**n**	Total number of data sets
**c1true**	Number of data sets for infants with IVH
**c2true**	Number of data sets for infants without IVH
**c1method**	Number of data sets classified by the ML-method as of infants with IVH
**c2method**	Number of data sets classified by the ML-method as of infants without IVH
**n1**	c1true × c1method/n
**n2**	c2true × c2method/n
**Expected accuracy (EA)**	(n1 + n2)/n
**Observed accuracy (OA)**	Number of correctly identified data sets/n
**Cohen’s kappa**	(OA-EA)/(1-EA)
**Mean accuracy**	Mean value of OA
**n1true**	Number of correctly identified data sets for infants with IVH
**n2true**	Number of correctly identified data sets for infants without IVH
**Sensitivity**	n1true/c1true
**Specificity**	n2true/c2true

Categorical characteristics (such as sex, multiple birth, normal vaginal delivery, and rupture of amniotic sac) were compared by their relative values in % along with p-value calculated by Fisher’s exact test. For continuous variables (such as gestational age, birth weight, and clinical measurements) mean values were compared using the two-sided Wilcoxon’s rank-sum test. A p-value < 0.05 was considered as statistically significant.

## Results

Table 5 lists the variables used for the construction of ML- models, their mean values (±standard deviation) computed over all available ​​records for each group, and p-values for the null hypothesis that the means for the control and affected groups are equal.

**Table 5:** Comparison of variable means for the control and affected groups. For the affected group only records taken before hemorrhage were considered for the model.

Variable	Extremely preterm (23–26 WG), n = 137			Very preterm (27–30 WG), n = 92		
	Control (n = 62)	Affected (n = 75)	p-value^a^	Control (n = 49)	Affected (n = 43)	p-value^a^
**A1**	5.57±1.93	5.45±1.69	0.057	6.41±2.04	5.85±2.03	<0.001
**A5**	6.94±1.60	7.05±1.52	0.059	7.86±1.13	7.30±1.24	<0.001
**A10**	7.98±1.31	7.92±0.91	<0.001	8.54±0.57	8.00±0.71	<0.001
**Ht [L/L]**	0.442±0.078	0.422±0.078	<0.001	0.467±0.080	0.458±0.088	0.495
**Thro [10**^**9**^**/L]**	198.48±80.48	180.28±75.00	0.002	206.21±90.9	165.82±78.4	<0.001
**Leu [10**^**9**^**/L]**	20.27±17.62	11.28±8.43	<0.001	9.12±5.58	10.84±9.41	0.128
**CRP [mg/dL]**	0.448±0.506	0.825±0.942	<0.001	0.646±0.828	1.55±3.705	0.007
**pH**	7.28±0.07	7.25±0.09	<0.001	7.32±0.06	7.27±0.09	<0.001
**pO**_**2**_ **[mmHg]**	43.07±12.52	47.73±14.39	<0.001	42.16±9.37	42.26±10.5	0.722
**SaO**_**2**_ **[%]**	91.56±4.39	90.27±4.33	<0.001	94.23±5.76	89.87±6.74	<0.001
**pCO**_**2**_ **[mmHg]**	47.65±9.49	48.40±11.18	0.389	44.09±7.61	48.65±9.05	<0.001
**MAP [mmHg]**	36.80±8.96	30.50±8.15	<0.001	40.45±8.58	37.16±7.58	<0.001
**CBF** **[ml/min/100g]**	10.94±5.43	8.89±4.79	<0.001	15.45±5.33	17.95±7.57	<0.001

^a^p-value for the null hypothesis that the group means for the control and affected groups are equal.

**Fig 1:**
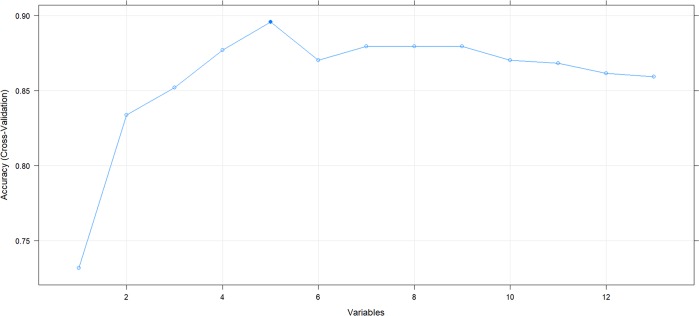
Optimal number of features in RF-models for extremely preterm infants with 23 to 26 WG.

**Fig 2:**
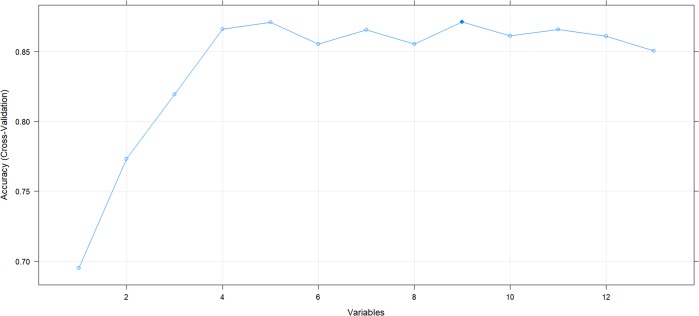
Optimal number of features in RF-models for very preterm infants with 27 to 30 WG.

The optimal number of features established for extremely preterm infants is 5 (Fig 1). For very preterm infants, although 9 attributes provided highest accuracy, 5 attributes yielded practically the same result (Fig 2).

**Fig 3:**
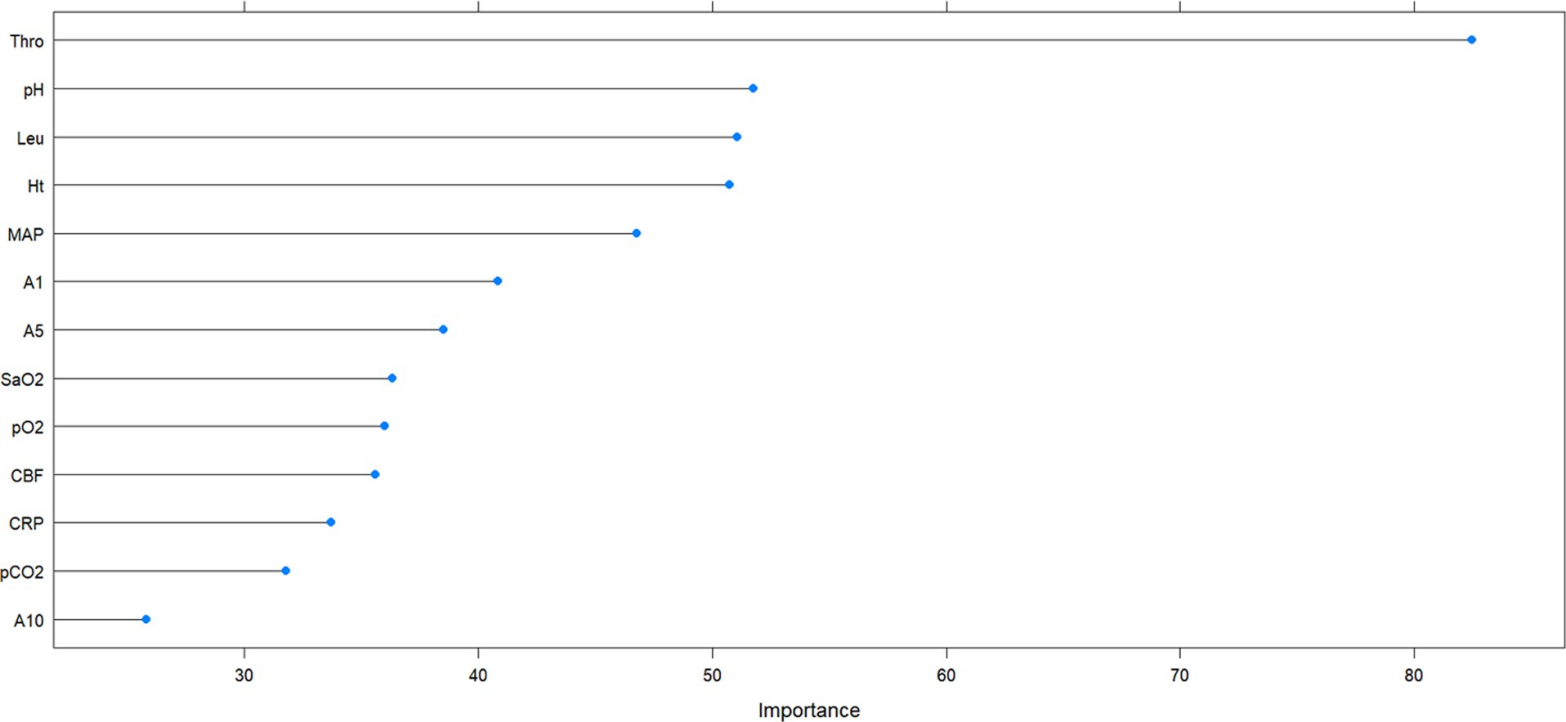
Variable importance plot for extremely preterm infants with 23 to 26 WG.

**Fig 4:**
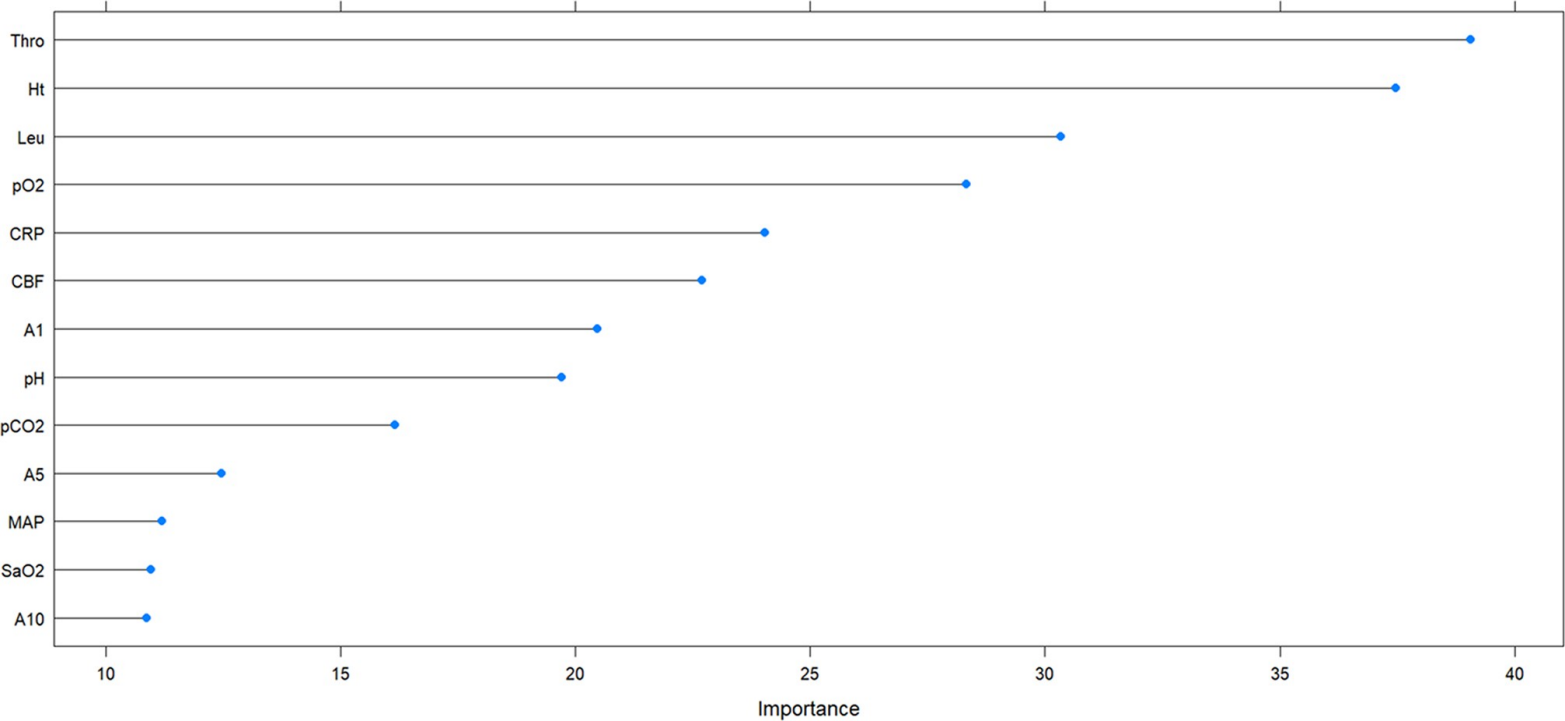
Variable importance plot for very preterm infants with 27 to 30 WG.

The importance of variables for the group of extremely and very preterm infants is plotted in Figs 3 and 4, respectively. To build ML-models, three top variables (Thro, pH, and Leu for extremely preterm and Thro, Ht, and Leu for very preterm infants) were supplemented by two variables chosen by enumerating all possible variants of the remaining 10 variables. Additionally, for each group, combinations of variables that were effective in the other group were also tested. Variables that were correlated were considered redundant and only one of them was used for each model. For extremely preterm infants, a correlation was established (the absolute value of correlation coefficient >0.5) between pCO_2_ and CBF, MAP and CBF, A1 and A5, A1 and A10, A5 and A10. For very preterm infants, in addition to these correlations, also correlations between Leu and CRP, CBF and CRP, pH and pCO_2_ were found.

Table 6 (extremely preterm infants) and Table 7 (very preterm infants) display the best results obtained. The first column in each table shows the variables used for the construction of the ML-model. The second column shows the mean accuracy of the obtained model and the third column Cohen’s kappa. The fourth column gives the accuracy of the model on unseen data; 95% confidence interval (CI) and the accuracy rate that can be achieved without a model are shown in the fifth and sixth columns, respectively. The seventh column shows a p-value indicating the probability of observing the same or better classification accuracy for the classifier with no information rate; the sensitivity and the specificity of the model are given in the eighth and ninth columns, respectively.

**Table 6:** Performance results for extremely preterm newborns.

Model variables	Mean accuracy	Mean kappa	Accuracy of prediction	95% CI	No information rate	p-value	Sensitivity	Specificity
**Thro, pH, Leu,** **Ht, A10**	0.877	0.735	0.89	(0.795, 0.952)	0.644	<0.001	0.923	0.872
**Thro, pH, Leu,** **Ht, A5**	0.910	0.807	0.918	(0.83, 0.969)	0.644	<0.001	0.923	0.915
**Thro, pH, Leu,** **Ht, A1**	0.913	0.812	0.959	(0.885, 0.991)	0.644	<0.001	0.923	0.979
**Thro, pH, Leu,** **Ht, CRP**	0.893	0.743	0.918	(0.804, 0.977)	0.714	<0.001	0.929	0.914
**Thro, pH, Leu,** **pO**_**2**_**, CRP**	0.861	0.664	0.918	(0.804, 0.977)	0.714	0.005	1.0	0.886
**Thro, Ht, Leu, CBF, A1**	0.879	0.747	0.872	(0.743, 0.952)	0.617	<0.001	0.889	0.862
**Thro, Ht, Leu,** **A1, SaO**_**2**_	0.889	0.756	0.883	(0.774, 0.952)	0.667	<0.001	0.9	0.875

**Table 7:** Performance results for very preterm newborns.

Model variables	Mean accuracy	Mean kappa	Accuracy of prediction	95% CI	No information rate	p-value	Sensitivity	Specificity
**Thro, Ht, Leu,** **pH, A5**	0.898	0.76	0.972	(0.855, 0.999)	0.694	3e-05	1.0	0.96
**Thro, Ht, Leu,** **pH, A1**	0.9	0.77	0.944	(0.813, 0.993)	0.694	0.0003	1.0	0.92
**Thro, Ht, Leu, pCO**_**2**_**, A5**	0.879	0.72	0.889	(0.739, 0.969)	0.694	0.006	0.818	0.92
**Thro, Ht, Leu, pCO**_**2**_**, A1**	0.892	0.752	0.944	(0.813, 0.993)	0.694	0.0003	1.0	0.92
**Thro, Ht, Leu, CBF, A1**	0.891	0.765	0.909	(0.708, 0.989)	0.636	0.004	0.875	0.929
**Thro, Ht, Leu,** **A1, SaO**_**2**_	0.864	0.676	0.931	(0.772, 0.992)	0.69	0.002	0.889	0.95

According to Table 6, for the group of extremely preterm infants, the most efficient sets of model variables included Thro, pH, Leu, Ht, A10, A5, A1, CRP, pO_2_, CBF, and SaO_2_.

For very preterm infants, the most effective predictors were Thro, Ht, Leu, pH, A1, A5, pCO_2_, CBF, and SaO_2_.

In both groups, prediction was most accurate for the sets of variables including Thro, Ht, Leu, pH, and one of the Apgar scores (A1 for extremely preterm and A5 for very preterm infants).

## Discussion

The occurrence of cerebral hemorrhage in preterm infants can cause lifelong disability. Its prevention is therefore an important aim.

ML-models constitute a promising tool in this endeavor through the identification of premature infants at risk for developing cerebral bleeding. The results on model performance presented above indicate that a good predictive ability can be achieved with different combinations of clinical parameters. The variables, Thro, Leu, Ht, pH, CBF, SaO_2_, A1, and A5 are common to the most effective sets of model variables, both for extremely as well as very preterm groups.

The association between these variables and the development of IVH in preterm neonates has been reported in several previous studies [22–31]. Occurrence of IVH is strongly associated with thrombocytopenia (lower number of thrombocytes in blood) [22], and, in very low birth weight infants, with an increase in leukocyte count [23]. Moreover, leukocyte count and CPR were found to be associated with histological chorioamnionitis [24], which in turn has also been identified as a risk factor of IVH [25]. Low initial Ht-levels in preterm infants with < 28 WG were shown to be associated with occurrence of IVH [26], with initial values of Ht < 45% having been associated with a 2-fold increase in cerebral hemorrhage probability in extremely low birth weight neonates [27]. According to [28], decreased values of pH together with hypercapnia (pCO_2_ > 45 mmHg) can also increase the risk of occurrence of IVH, and fluctuations of CBF can increase the risk of cerebral hemorrhage [29]. Although there is no direct evidence for an association between oxygen saturation and IVH, the reduced supply of oxygen may lead to an elevation of CBF in an attempt to maintain the levels of SaO_2_. Apgar scores are used as indicators of adverse outcome, including severe IVH [30, 31].

Machine learning methods were here used to determine the combinations of clinical variables that can most effectively identify infants at risk for cerebral bleeding. For extremely preterm infants, almost all of the most effective combinations of variables considered here included Thro, pH, and Leu. The other two variables were combinations of Ht, A1, A5, CBF, CRP, SaO_2_, and pO_2_. For very preterm neonates, the variables Thro, Ht, and Leu were always present in the most effective combinations, being supplemented by two variables from the following list: pH, A1, A5, CBF, SaO_2_, and pCO_2_. The absence of CRP in this list compared to that for extremely preterms can be explained by the correlation between CRP and Leu observed, such that one of the variables is redundant for the identification process in this population. This difference in the predictive role of CRP between extremely and very preterm neonates may reflect the reported influence of gestational age on CRP-levels during the first few weeks after birth [32].

**Fig 5:**
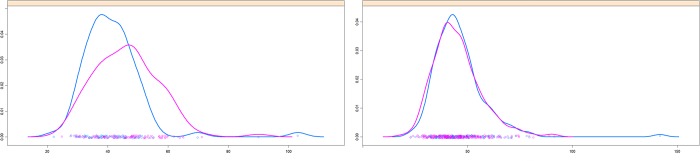
**Probability density functions for pO**_**2**_**-records of affected (pink curves) and control (blue curves) patients.** Left panel: Extremely preterm infants. Right panel: Very preterm infants.

**Fig 6:**
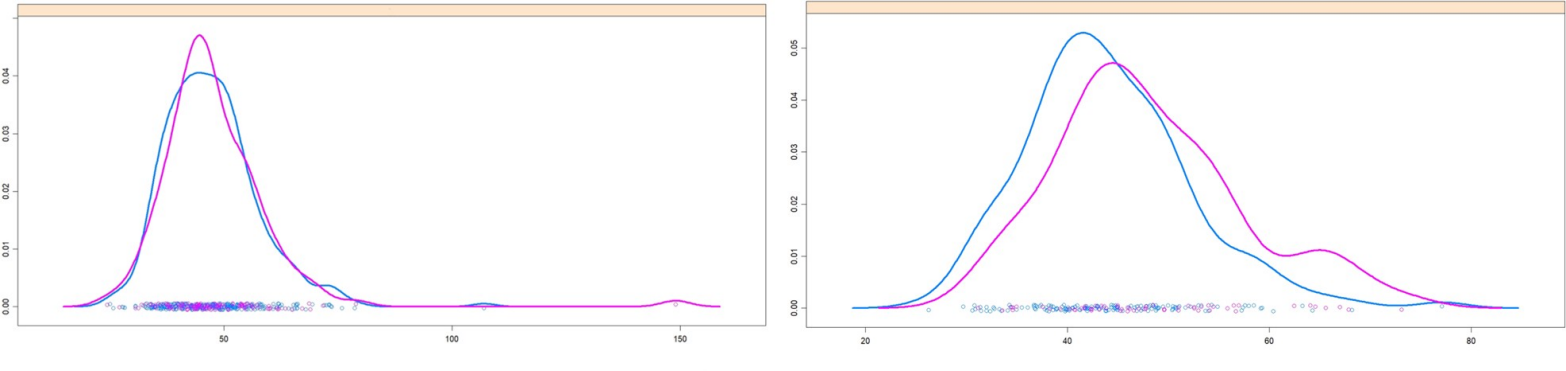
**Probability density functions for pCO**_**2**_**-records of affected (pink curves) and control (blue curves) patients.** Left panel: Extremely preterm infants. Right panel: Very preterm infants.

It is also worth to mention that the variable pO_2_ is present in the sets of model variables for extremely preterm but not for very preterm infants. This feature can be used to distinguish controls from affected extremely preterm infants (see Table 6). Fig 5 illustrates the difference in probability distribution of pO_2_-records between the control and affected groups for extremely (left panel) and very preterm (right panel) infants. The clearly different distributions of pO_2_ between control and affected extremely preterms, with a higher average pO_2_ value for the latter group (see Table 5), may reflect the recommended management of extremely preterm neonates, which targets towards high (91–95%) arterial oxygen saturation values [33, 34]. Whilst this is aimed to reduce mortality, the rate of occurrence of other complications has been observed not to change with the arterial oxygen saturation values. By contrast, very preterm infants with 27 to 30 WG are better distinguished by pCO_2_ than extremely preterm infants, as indicated by the presence of pCO_2_ in the sets of variables for this age group (Table 7). Fig 6 shows the difference in probability distribution of pCO_2_-records between the control and affected group for extremely (left panel) and very preterm (right panel) infants. This change in the set of parameters that allow a better separation between control and affected groups may reflect differences in development, for example of the lung function, between extremely and very preterm neonates. Although short, this period of time that separates extremely and very preterm infants might be sufficient to provide the very preterms a more developed condition relative to the extremely preterms, such that they do not need the same O_2_ ventilation support. However, relative to controls of the same age group, infants in the affected very preterm group may still have a less effective breathing function, that reflects in differences in the levels of CO_2_ in the blood relative to controls. Further clinical work and further development of the machine-learning models are however needed to be able to clarify the differences found.

The machine-learning models here presented were built upon laboratory variables that, apart from CBF, are currently collected during routine clinical monitoring. For example, variations in blood pressure may lead to hypoperfusion and hyperperfusion of the brain and may require pharmacological treatment. In case of hypoperfusion cardiovascular therapy with volume loading and pharmacological therapy are often recommended. And whilst changes in specific parameters require specific treatments, there are however no definitive or strict recommendations. By considering several clinical factors, instead of analyzing effects of individual or of a small group of parameters, the machine-learning models presented account for the possibility of brain bleeding involving the combination of several factors. Detecting such multidimensional higher-risk conditions is not straightforward for a person in a clinical setting, but may be facilitated by model-supported computations.

In addition to the above, by helping to identify preterms at risk of brain bleeding, the machine-learning models presented may promote a close monitoring of these preterms earlier than currently is the case and this way allow for an earlier intervention and control of the development of clinical parameters, ultrasound of brain and of noticeable neurological problems.

The fact that several models with a good predictive ability were obtained both for extremely and very preterm infants makes it possible to use those models for which the greatest amount of necessary data is available when assessing the risk of cerebral hemorrhage in an individual neonate. In addition, conclusions about the individual risk of hemorrhage can be made based on testing results for several models, focusing on the worst case.

In addition to the laboratory variables the ML-models presented were based also on information about the CBF, for different combinations of MAP and pCO_2_ values. These are also important to evaluate the risk of brain bleeding. CBF values cannot however be obtained experimentally (with standard clinical measures) but need to be calculated via modelling [9].

Let us mention some limitations of the presented research. 1) Since data wеrе collected retrospectively, patients had a different number of measurements. 2) The control and affected groups were not completely matched by sex but this factor was not included in the models. The objective difficulty of matching by sex lies in the fact that male preterm neonates have a higher risk of IVH compared to female preterm neonates [35, 36]. 3) Although only measurements up to the day when the previous planned ultrasound examination took place were input into the model (Table 2), some measurements may have been included that in reality were made already after the bleeding. This is because the exact time of bleeding could not be exactly determined. For example, IVH could happen on the first day of life just after the first ultrasound examination, but be diagnosed on the second or third day of life. Nevertheless, all the measurements made on the first day were included. However, we expect the number of such “after-bleeding” measurements is small in comparison with the number of “before-bleeding” measurements and not to significantly influence the results obtained. 4) The data were collected retrospectively over a ten year period (2006–2016) during which changes in the management of preterm infants could have occurred.

Though the RF-models employed showed good performance, in future work we will plan to test other machine learning algorithms (e.g. kNN, SVM, and CART), considering additional features. The developed models are planned to be tested against new data. To this end, additional data will need to be collected and input into the models. Additional clinical records will enable the further development of ML-models for the different ages for which the measurements were obtained, which will contribute to improve the predictive power of the models. We expect the models to be developed may help neonatologists in the future to timely identify infants at risk of cerebral hemorrhage and in this way reduce the rate of its occurrence and of consequent impairments.
